# Alterations in semen parameters in men wıth epilepsy treated with valproate

**Published:** 2015-07-06

**Authors:** Hatice Kose-Ozlece, Faik Ilık, Kursat Cecen, Nergiz Huseyınoglu, Ataman Serım

**Affiliations:** 1 Department of Neurology, School of Medicine, Kafkas University Medical Faculty, Kars, Turkey; 2 Department of Neurology, School of Medicine, Mevlana University, Konya, Turkey; 3 Department of Urology, School of Medicine, Kafkas University Medical Faculty, Kars, Turkey

**Keywords:** Valproate, Male Infertility, Epilepsy, Antiepileptic Drugs, Reproductive Dysfunction

## Abstract

**Background:** Besides the well-known adverse effects of valproate (VPA), disorders related to male reproductive functions have been reported. Furthermore, only a limited number of previous studies have reported the relationship between VPA dose and impairment of the hormonal axis and semen quality. A patient with reversible changes that occurred in the sperm parameters after a dose increment of VPA.

**Methods: **A 34-year-old male patient who was diagnosed with juvenile myoclonic epilepsy almost 15 years ago was admitted to our clinic. His seizures responded well to high doses of VPA treatment.

**Results: **As the VPA dose was increased, consecutive semen analyses were performed and averaged for each dose; the results showed a remarkable decline in the sperm count and a manifest loss of sperm motility. VPA treatment was gradually diminished and stopped; meanwhile, treatment with another antiepileptic (lamotrigin) was initiated to control the patient’s seizures. Nine months later, the patient’s semen analysis was within normal ranges. After modification of the patient’s treatment regimen, he and his wife had a healthy baby.

**Conclusion:** We suggest that VPA-dependent impairments in the hormone and semen analysis parameters were reversible after the termination of medical treatment, and that the VPA treatment did not cause permanent hormonal deregulation and, these side effects are dose dependent.

## Introduction

In the population of epileptic male patients, dysregulation of the gonadotropic hormones, impairments of semen analysis parameters, sexual dysfunctions and a decline in fertility capacity have been reported. One probable cause of the reduction in fertility capacity, which has been reported in some previous studies, is the effect of antiepileptic drugs.^1^

Valproate (VPA) is a broad spectrum antiepileptic agent which is effective in the treatment of many types of generalized and partial seizures. It has proven to be especially effective in treating primary generalized, tonic-clonic, myoclonic, and absence seizures; furthermore, it can be successfully used to treat juvenile myoclonic epilepsies where all of the previously mentioned seizure types may occur.^2^

Gastrointestinal side effects, weight gain, abnormalities in blood parameters, tremor, sedation, hair loss, and impairment in liver function are some of the most frequent adverse effects encountered during VPA treatment. Besides the aforementioned adverse effects, disorders related to male reproductive functions have been reported. Alterations in the hormonal axis and impairments in semen parameters have been especially emphasized.^3^^-^^5^ A limited number of previous studies have reported a relationship between VPA dose and impairment of the hormonal axis and semen quality.^6^

This case describes reversible changes that occurred in the sperm parameters after a dose increment of VPA in a male patient with a diagnosis of juvenile myoclonic epilepsy.

## Materials and Methods

A 34-year-old male patient who was diagnosed with juvenile myoclonic epilepsy almost 15 years ago was admitted to our clinic for follow-up; his main complaint was an increase in the frequency of his seizures. Myoclonic jerks were symmetrically and involved the arms. Previously, his seizures responded well to high doses of VPA treatment. Subsequently, his medication doses were gradually reduced under the supervision of his doctor. He had been receiving 500 mg/day of VPA for 6 years. His dose was upgraded to 1500 mg/day, and seizure control was achieved.

The patient had been married for almost 7 years and had regular follow-ups at a urology clinic for the last 3 years due to infertility issues. At the moment, his spermiogram parameters were within normal ranges, although close to the lower cut-off values. His wife was healthy and her reproductive function was completely normal.

## Results

As the VPA dose was increased, consecutive semen analyses were performed and averaged for each dose; the results showed a remarkable decline in the sperm count and a manifest loss of sperm motility. In addition to these impairments in the semen analysis, anomalies in the sperm morphology were also reported (Table 1).

The patient’s hemogram and routine biochemical parameters were in the normal range. Follicle-stimulating hormone (FSH) and luteinizing hormone **(**LH) measurements in the serum of the patient were below the cut-off value; on the other hand, dehydroepiandrosterone and testosterone measurements were above the normal range. The patient was consulted to the urology department for further evaluation and exclusion of other causes of infertility (obstruction, testicular infection, febrile disease in the recent past, and use of another drug). The semen for infertility research was obtained and analyzed using World Health Organization (WHO) guidelines and sperm morphology was performed using teiberg criteria.^7^^-^^9^ After a differential diagnosis accounting for other possible causes of infertility, the patient’s condition was diagnosed as oligoasthenospermia when VPA dose is 1000 mg/day and then azoospermia when dose is 1500 mg/day related to VPA. VPA treatment was gradually diminished and stopped; meanwhile, treatment with another antiepileptic (lamotrigin) was initiated to control the patient’s seizures. 9 months later the patient’s semen analysis was within normal ranges.

20 months after modification of the patient’s treatment regimen, he and his wife had a healthy baby. 

## Discussion

VPA is a wide-spectrum and successful antiepileptic agent used to treat many different types of epileptic seizures; it is also frequently used for medical conditions apart from epileptic seizures.^2^

The underlying mechanism of VPA that affects the male reproductive system is not yet fully understood. One of the most important suggested mechanisms is oxidative stress. Reactive oxygen species production can cause direct damage to DNA, proteins or lipids and/or by altering signal transduction of gene expressions. It is well known that VPA act as histone deacetylase inhibitor and modulate several gene expressions by histone hyperacetylation. The histones have multiple post-translational modifications, which are critical to the regulation of spermatogenesis.^10^ On the other hand, the histone to protamine transition is the major step for healthy spermatogenesis.^11^ Protamines are the important nuclear proteins in sperm cells. These proteins provide the correct packaging of the paternal DNA. Many studies have found that abnormal changes of protamine expressions leading to male infertility.^12^^-^^13^

Another suggested mechanism is the inhibition of the liver enzymes, which results in a decline in the estradiol level. This hypothesis suggests that decreasing estradiol levels in the blood have a negative feedback effect on the hypothalamus, and via the hypothalamohypophyseal axis, it affects the hypophyseal gland.^14^

**Table 1:** Patient’s consecutive semen parameters results

**Valproate dose (mg/day)**	**500 ** **(for 6 year)**	**1000 ** **(for 4 months)**	**1500 ** **(for 2 year)**	**9 months after stopped ** **the treatment**
Valproate level (μg/ml)	21	61	109	< 3
Sperm concentration (10^6^/ml)	87	2	5,1	91,2
Sperm viability (%)	55	20	20	70
Progressive motility (%)	25	5	5	35
Total motility (%)	42	10	10	60
Normal morphology (Tygerberg criteria) (%)	8	1	0	28

Another hypothesis asserts that VPA causes impairment to the serotonergic and GABAergic steroid metabolisms, resulting in an increase in dehydroepiandrostenedione sulfate (DHEAS) concentration in the blood. An increase in DHEAS concentration triggers a decrease in the hypophyseal hormones such as LH and FSH and this decrement in hypophyseal hormones manifests clinically as a reproductive disorder.^14^ In our case, the patient’s testosterone levels were above the normal range; on the other hand, LH and FSH levels were below the normal value.

In many previous studies, it has been reported that sperm analysis abnormalities can be seen in patients under treatment with VPA.^1^ In a study conducted by Roste et al., comparing the effects of VPA and carbamazepine treatments on sperm analysis parameters, it was found that sperm tail abnormalities were significantly higher in patients receiving VPA treatment^1^ And Roste et al. demonstrated men on VPA also had significantly lower carnitine levels, which may have implications for sperm motility.^15^ In another study, Isojarvi et al. reported a higher risk of sperm motility disorders and a higher chance of encountering morphological abnormalities in patients under the treatment with VPA.^4^ Although VPA’s mechanism of action on the sperm is not fully understood, in in vitro research it has been proposed that VPA directly affects sperm motility by inducing membrane stabilization.^16^

In our case, the patient’s sperm analysis parameters were in normal ranges, close to the lower cut-off values, when he was receiving 500 mg/day of VPA. An increase in the VPA daily dose was accompanied by a further impairment in semen analysis parameters. These results led us to think that the side effects of VPA are dose dependent. In a study comparing rats receiving low and high doses of VPA, rats under treatment with high dose VPA were observed to have a significant loss of testicular mass and also a severe grade of testicular atrophy.^6^

In our case, because of the potential side effects of the VPA treatment, the dose was diminished and finally cut off. After almost 9 months from the termination of VPA treatment, sperm analysis parameters were in normal ranges; sperm count, motility and morphology studies showed results within normal ranges, and afterward the patient fathered a healthy baby. Similar normalization of sperm analysis results and successful fertilization have been reported in previous case reports and clinical research.^17^^,^^18^ We suggest that VPA-dependent impairments in the hormone and semen analysis parameters were reversible after the termination of medical treatment, and that the VPA treatment did not cause permanent hormonal deregulation.

## Conclusion

Finally, in chronic medical conditions such as epilepsy where patients have to receive medical treatment for prolonged periods of time, we have to be careful about the potential side effects of these drugs. Especially in epileptic patients admitted to clinics with infertility disorders, we have to be careful about the selection of medical treatment and, if necessary, alternative medical treatments can be tried.

## Notes:

**How to cite this article: **Kose-Ozlece H, Ilık F, Cecen K, Huseyınoglu N, Serım A. Alterations in semen parameters in men wıth epilepsy treated with valproate. Iran J Neurol 2015; 14(3): 164-7.
